# Supplementary material for: Antibiotic Resistance in Wastewater Treatment Plants and Transmission Risks for Employees and Residents: The Concept of the AWARE Study
Source: Antibiotics (Basel). 2021 Apr 21;10(5):478. doi: 10.3390/antibiotics10050478 (PMC8143112; doi:10.3390/antibiotics10050478)
Supplement: Supplementary file 1 [file antibiotics-10-00478-s001.zip › S2_AWARE_questionnaire_Operators.pdf]

LMU KLINIKUM
DER UNIVERSITÄT MÜNCHEN

CAMPUS INNENSTADT

INSTITUT UND POLIKLINIK FÜR
ARBEITS-, SOZIAL- U. UMWELTMEDIZIN

Questionnaire

Antibiotic Resistance in Wastewater: Transmission Risks for Employees and Residents around Wastewater Treatment Plants

OCCUPATIONAL AND ENVIRONMENTAL EPIDEMIOLOGY & NET-TEACHING UNIT

Head: PROF. DR. KATJA RADON, MSC

AWARE

Study team: Dr. Laura Wengenroth, Dr. Tobias Weinmann
Institute and Outpatient Clinic for Occupational, Social and Environmental Medicine
Clinical Centre of the Ludwig Maximilian University (LMU)
Ziemssenstr. 1, 80336 München
Telephone: 089/4400-52483
Telefax: 089/4400-54954
E-mail: arb.aware@med.uni-muenchen.de

Study-ID:

Dear participant,

thank you for your interest in participating in the AWARE study. In this questionnaire we will ask you several questions about you, your work and family environment and your health. Please answer as completely and accurately as possible. If you are unsure about a question, please select "no". Filling out the questionnaire should take you around 20 minutes.

Thank you very much!

Your AWARE-Team

Contact
Dr. Laura Wengenroth, Dr. Tobias Weinmann

Tel.: 089 4400-52483 · Fax: 089 4400-54954
E-Mail: arb.aware@med.uni-muenchen.de
Website: www.aware-study.eu

Klinikum der Universität München
Ziemssenstr. 1
80336 München

Postal address: Postfach 15 20 25 · 80051 München

Study Coordination

Prof. Dr. Katja Radon · Institute and Outpatient Clinic for Occupational, Social and Environmental Medicine, Clinical Centre of the Ludwig Maximilian University (LMU) Munich, Germany

Prof. Dr. Ana Maria de Roda Husman · National Institute for Public Health and the Environment (RIVM), Bilthoven, Netherlands

Prof. Dr. Carmen Chifiriuc · Research Institute of the University of Bucharest (ICUB), Bucharest, Romania

Prof. Dr. Joakim Larsson · Institute of Biomedicine, University of Gothenborg, Göteborg, Sweden

Please follow the following instructions to help you fill out the questionnaire:

Please mark your answer to each question by marking the area inside the answer box with a cross as shown in the example:

Example: ☒

If you make a mistake and need to correct it, please fill in the complete box:

Example: ■

To answer open questions, please write clearly and in block letters on the corresponding row. If a number is required, please write it clearly in the corresponding field:

Example: 16 years

Please make sure to answer all questions by going line by line. You may skip questions only when the text explicitly indicates it.

No ☒ ………….. → Please continue with question XY

Yes ………… ☐

If you mark „yes“, please continue on with the next question. If you mark „no“, please proceed only to the question indicated by the arrow!

Please check again for completeness after you have answered the questionnaire.

GENERAL INFORMATION ON WWTP

1. **Is the amount of inhabitants served known?**
   ☐ No → Please continue with question 3
   ☐ Yes

2. **If yes, how many inhabitants does the wastewater treatment plant serve?**
   I__I__I__I__I__I__I__I inhabitants

3. **What is the typical daily flow (dry weather conditions)?**
   I__I__I__I__I__I__I m^3/day

4. **What is the maximal daily flow (wet weather conditions)?**
   I__I__I__I__I__I__I m^3/hour

5. **What is the design capacity in population equivalents?**
   ____________________p.e.
   Population equivalent (p.e.) *is a term used to measure the organic biodegradable load generated in an urban area. It takes into account the load generated by the resident population, the non-resident population (e.g. tourists) and industries. A population equivalent of 1 is definfed as the organic biodegradable load having a fiveday biochemical oxygen demand of 60 g of oxygen per day or 120 g COD/day*
   COD = chemical oxygen demand

6. **What is the actual utilization (in population equivalents p.e.)?**
   ____________________p.e.

7. **What is the food to microorganism ratio (F/M-ratio) in g BOD/kg d.s./day?**
   ____________________ g BOD/kg d.s./day
   BOD *= biochemical oxygen demand*
   d.s. = dry solids

8. **On which biological method is the treatment based?**
   ☐ Activated sludge process → Please continue with question 10
   ☐ Others

9. **On which other biological methods is the treatment based?**

   ____

10. **Does this WWTP treat hospital wastewater?**
    ☐ No → Please continue with question 81
    ☐ Yes
    ☐ Do not know → Please continue with question 81

11. **If yes, from how many hospitals?**
    I__I__I hospitals

12. **Does this WWTP treat wastewater from nursing homes / elderly peoples' homes?**

☐ No → Please continue with question 14
☐ Yes
☐ Do not know → Please continue with question 14

13. If yes, from how many nursing homes / elderly peoples' homes?
|__|__|__|

14. Is the following treatment existing in the plant: Primary sedimentation?
☐ No
☐ Yes

15. Is the following treatment existing in the plant: Aeration, biological stages?
☐ No → Please continue with question 17
☐ Yes

16. How many biological stages exist?
☐ One stage
☐ Two stages

17. Is the following treatment existing in the plant: Nitrification?
☐ No
☐ Yes

18. Is the following treatment existing in the plant: Denitrification?
☐ No
☐ Yes

19. Is the following treatment existing in the plant: Phosphorus removal?
☐ No → Please continue with question 21
☐ Yes

20. Does phosphorous removal take place chemically or biologically?
☐ Chemically
☐ Biologically
☐ Chemically and biologically

21. Is the following treatment existing in the plant: Solid separation?
☐ No
☐ Yes

22. Is the following treatment existing in the plant: Advanced treatment such as disinfection of effluent or removal of micropollutants?
☐ No → Please continue with question 24
☐ Yes

23. Which advanced treatment for desinfection of effluent or removal of micropollutants do you use?

____

24. Is the following treatment existing in the plant: Open sludge storage?

☐ No
☐ Yes

25. Sludge treatment is present for the following streams:

☐ Sludge thickening
☐ Sludge digestion
☐ Sludge dewatering

26. Are the following wastewater treatment facilities present at the WWTP: Air release valve or manhole?

☐ No
☐ Yes

27. Are the following wastewater treatment facilities present at the WWTP: Pumping station?

☐ No → Please continue with question 29
☐ Yes

28. Is it a wet well or dry well pumping station?

☐ Wet well
☐ Dry well

29. Are the following wastewater treatment facilities present at the WWTP: Septage receiving and handling facilities?

☐ No
☐ Yes

30. Are the following wastewater treatment facilities present at the WWTP: Side stream returns (including digester decant, dewatering return flows, or backwash water)?

☐ No
☐ Yes

31. Are the following wastewater treatment facilities present at the WWTP: Return sludge?

☐ No → Please continue with question 33
☐ Yes

32. How is return sludge transported?

☐ Pump
☐ Headworks

33. Are the following wastewater treatment facilities present at the WWTP? Are these open or closed: Headworks?

"Open" means that residents may experience odor nuisance, while "closed" means that residents can not experience any odor nuisance.

☐ No

☐ Yes, open
☐ Yes, enclosed

34. Are the following wastewater treatment facilities present at the WWTP? Are these open or closed: <u>Screenning</u>?
☐ No
☐ Yes, open
☐ Yes, enclosed

35. Are the following wastewater treatment facilities present at the WWTP? Are these open or closed: <u>Pre-aeration</u>?
☐ No
☐ Yes, open
☐ Yes, enclosed

36. Are the following wastewater treatment facilities present at the WWTP? Are these open or closed: <u>Grit removal</u>?
☐ No
☐ Yes, open
☐ Yes, enclosed

37. Are the following wastewater treatment facilities present at the WWTP? Are these open or closed: <u>Flow equalization</u>?
☐ No
☐ Yes, open
☐ Yes, enclosed

38. Are the following wastewater treatment facilities present at the WWTP? Are these open or closed: <u>Primary Clarifier</u>?
☐ No
☐ Yes, open
☐ Yes, enclosed

39. Are the following wastewater treatment facilities present at the WWTP? Are these open or closed: <u>Aeration Basin</u>?
☐ No → Please continue with question 42
☐ Yes, open
☐ Yes, enclosed

40. How does wastewater treatment take place in the aeration basin?
☐ Anoxic
☐ Aerobic
☐ Anaerobic

41. How does aeration take place in the aeration basin?
☐ Cascade
☐ Vertical axis surface aerator
☐ Fine bubble aeration
☐ Frush aeration
☐ Other

42. **Are the following wastewater treatment facilities present at the WWTP? Are these open or closed: <u>Trickling filter</u>?**
☐ No
☐ Yes, open
☐ Yes, enclosed

43. **Are the following wastewater treatment facilities present at the WWTP? Are these open or closed: <u>Secondary Clarifier</u>?**
☐ No
☐ Yes, open
☐ Yes, enclosed

44. **Are the following sludge and biosolids facilities present at the WWTP? Are these open or closed?**

"Open" means that residents may experience odor nuisance, while "closed" means that residents can not experience any odor nuisance.

	**No**	**Yes, open**	**Yes, enclosed**
Sludge thickening	☐	☐	☐
Sludge storage after mechanical dewatering	☐	☐	☐
Composting facilities	☐	☐	☐
Sludge drying bed	☐	☐	☐

45. **Are the following sludge and biosolids facilities present at the WWTP: <u>Wet sludge storage</u>?**
☐ No → Please continue with question 47
☐ Yes

46. **Is the wet sludge storage covered or uncovered?**
☐ Covered
☐ Uncovered

47. **Are the following sludge and biosolids facilities present at the WWTP: <u>Wet sludge loadout facilities</u>?**
☐ No → Please continue with question 49
☐ Yes

48. **Are the wet sludge ladaout facilities covered or uncovered?**
☐ Covered
☐ Uncovered

49. **Are the following sludge and biosolids facilities present at the WWTP: <u>Digestion</u>?**
☐ No → Please continue with question 51
☐ Yes

50. **How does digestion take place?**
   - ☐ Aerobic
   - ☐ Anaerobic, mesophilic
   - ☐ Anaerobic, thermophilic

51. **Are the following sludge and biosolids facilities present at the WWTP: <u>Mechanical Dewatering</u>?**
   - ☐ No → Please continue with question 53
   - ☐ Yes

52. **How does mechanical dewatering take place?**
   - ☐ Belt filter press
   - ☐ Recessed plate filter press
   - ☐ Centrifuge
   - ☐ Other

53. **Are the following sludge and biosolids facilities present at the WWTP: <u>Dry sludge loadout facilities</u>?**
   - ☐ No → Please continue with question 55
   - ☐ Yes

54. **Are the dry sludge loadout facilities covered or uncovered?**
   - ☐ Covered
   - ☐ Uncovered

55. **Are the following sludge and biosolids facilities present at the WWTP: <u>Alkaline stabilization (stabalized solids generation resulting from reaction with lime)</u>?**
   - ☐ No
   - ☐ Yes

Thank you very much for participating in the AWARE Study!

With your help we came closer to achieving our goals of the study.

If you have any further questions, please do not hesitate to contact us via our webpage www.aware-study.eu or via email arb.aware@med.uni-muenchen.de.

Here, you have the possibility to make comments:

Dr. Laura Wengenroth, Dr. Tobias Weinmann
Institute and Outpatient Clinic for Occupational, Social and Environmental Medicine
Clinical Centre of the Ludwig Maximilian University (LMU)
Ziemssenstr. 1, 80336 München
Telephone: 089/4400-52483
Telefax: 089/4400-54954
E-mail: arb.aware@med.uni-muenchen.de
